# Editorial: Computational accounts of reinforcement learning and decision making in psychiatric disorders

**DOI:** 10.3389/fpsyt.2022.966369

**Published:** 2022-07-25

**Authors:** Henry W. Chase, Robert C. Wilson, James A. Waltz

**Affiliations:** ^1^Department of Psychiatry, University of Pittsburgh School of Medicine, Pittsburgh, PA, United States; ^2^Department of Psychology, University of Arizona, Tucson, AZ, United States; ^3^Maryland Psychiatric Research Center, University of Maryland School of Medicine, Baltimore, MD, United States

**Keywords:** computational psychiatry, reinforcement learning, Hierarchical Gaussian Filter, value-based decision making, information seeking

Many psychiatric disorders are associated with aberrations of decision making (1). As well as having implications for patients' quality of life, such differences may be indicative of alterations in neural systems which underlie the representation of value. A definition of the “computational” level of analysis (2) is centered on the broad objectives that a system is seeking to achieve. In the case of valuation, organisms, including humans, are pre-occupied with making choices that help the individual, or the social group of which it is a part, survive (3). This involves the pursuit of reward (e.g., nutrition), and avoidance of threats (e.g., predators) or costs (e.g., effort). Algorithmic models of reinforcement learning (4) describing behavior in these terms have received extensive support across species and methodologies (5). In this special issue, four groups of authors examine mechanisms of the acquisition and expression of value across a range of different psychiatric conditions: schizophrenia, depression, anxiety, impulsivity, and opiate addiction. The articles employ a variety of computational modeling approaches, including reinforcement learning, utility-based decision-making models, and Bayesian models.

Alvarez et al. examined decision making across risky, delayed, and ambiguous options within the context of a longitudinal design of opiate use disorder and healthy control participants employing daily smartphone assessments. The authors found that opiate use disorder patients demonstrated greater delay discounting vs. healthy control participants, but no differences in risk or ambiguity tolerance. In the patients, preference for risky options, but not delay or ambiguity, increased with more positive mood. A key contribution of this work is to demonstrate that an individual's choice preferences may not be fixed, but may be coupled to variation in mood. As well as having implications for how option values are constructed, these findings potentially have crucial clinical implications regarding the identification of high-risk states which might predict relapse (6).

Zou et al. describe probabilistic reversal learning task performance across variation in self-reported impulsivity, employing reinforcement learning, and Bayesian inferential models. They found that participants reporting relatively higher levels of impulsivity showed an increased likelihood of switching after particular sequences of feedback that were characterized by sequential punishment. The finding may reflect a reduced ability in impulsive individuals to adopt consistent and adaptive long-term policies in the face of negative feedback. Strikingly, individual differences in impulsivity were not reflected in model parameters obtained from either model, suggesting the need to consider new modeling strategies for such tasks.

Smith et al. report on a study designed to distinguish directed from random exploration, using the “Horizon Task.” Although balancing exploration (information seeking) with exploiting known sources of reward is an important consideration in reinforcement learning (4), few paradigms are capable of distinguishing behavior specifically elicited to reduce uncertainty about a stimulus or the environment (directed exploration), from an undirected reduction in exploitation which also affords opportunities to learn about the environment (random exploration). The authors showed that directed exploration is reduced in individuals with higher levels of depression and anxiety symptoms, but increased with greater levels of self-reported cognitive reflection. Further analysis suggested that the preferences shown by more depressed/anxious individuals might be explained by increased ambiguity aversion. In general, information seeking has been relatively under-evaluated in the context of psychiatric disorders, although it may receive further attention with the development of active inference models (7).

Katthagen et al. provided a comprehensive review of 17 studies involving the application of dynamic belief updating models to the analysis of choice behavior in learning paradigms in psychosis. This review considered a variety of models including reinforcement learning, and also Hierarchical Gaussian Filter and Change Point Detection which both approximate Bayesian inference. Key concepts here are the representation of the volatility of the environment and the contingency between a given cue and outcome. In a volatile environment, the cue/outcome contingency can change substantially. Models reviewed by Katthagen et al. show adaptive learning rates which can accommodate such changes. Overall, the authors concluded that an overestimation of volatility, and mis-calibrated belief updating, are consistent findings within the schizophrenia literature.

As computational models of learning and choice become more widely adopted for understanding psychiatric illness, we might consider some general implications of this approach as exemplified by the articles in this special issue.

First, as described, the theoretical basis of these articles in translational research suggests the potential for cross-species integration and as well-mapping across levels of abstraction (e.g., evaluating causal interventions in experimental animals). An example here might be the Pearce-Hall model (8) introduced by Katthagen et al. Such translational considerations may encourage the use of paradigms with clear theoretical parallels across species (9).

Second, an overall theme of the articles is the emphasis on *similarities* across different psychiatric disorders, insofar as investigations of quite divergent patient groups are united by the sensitivity of value-based decision-making tasks to clinically-relevant individual differences. Of course, there are numerous, salient distinctions between the exact approaches taken by each group, but the potential for a common set of computational principles relating to valuation that might be relevant across a wide range of patient groups is implied. This broadly accords with the Research Domain Criteria [RDoC (10)] approach, in which constructs such as reward responsiveness or threat sensitivity can show substantial variation across different diagnostic subgroups, with similar consequences in each.

Third, the articles highlight several important considerations for model development, including: (1) the manner in which learning and decision making are modulated by uncertainty (Katthagen et al.; Smith et al.), (2) the distinction between explicit and implicit learning (Smith et al.), (3) the importance of capturing within-subject variation (Alvarez et al.), and (4) a central role for model comparison and for empirical data in model development (Zou et al.; Katthagen et al.).

In summary, the variety of methods and principles employed by the articles, and the breadth of implications for psychiatry, reveal the growing vitality of this field of research. Further consideration of these principles should contribute to the emergence of the field of computational psychiatry and increase our understanding of mental disorders.

## Author contributions

HWC wrote the initial draft of the manuscript. All authors developed the conceptual basis of the manuscript and then reviewed and edited it. All authors contributed to the article and approved the submitted version.

## Conflict of interest

The authors declare that the research was conducted in the absence of any commercial or financial relationships that could be construed as a potential conflict of interest.

## Publisher's note

All claims expressed in this article are solely those of the authors and do not necessarily represent those of their affiliated organizations, or those of the publisher, the editors and the reviewers. Any product that may be evaluated in this article, or claim that may be made by its manufacturer, is not guaranteed or endorsed by the publisher.
